# Paramagnetic Sensors for the Determination of Oxygen Concentration in Gas Mixtures

**DOI:** 10.1021/acssensors.2c00938

**Published:** 2022-10-27

**Authors:** Krzysztof Jasek, Mateusz Pasternak, Michał Grabka

**Affiliations:** † Faculty of Advanced Technologies and Chemistry, Military University of Technology, Warsaw00-908, Poland; ‡ Faculty of Electronics, Military University of Technology, Warsaw00-908, Poland

**Keywords:** oxygen sensor, magnetic gas sensor, magnetoacoustic sensors, Pauling cell, paramagnetism, paramagnetic gas, deflection of the paramagnetic gas stream, thermo magnetic wind

## Abstract

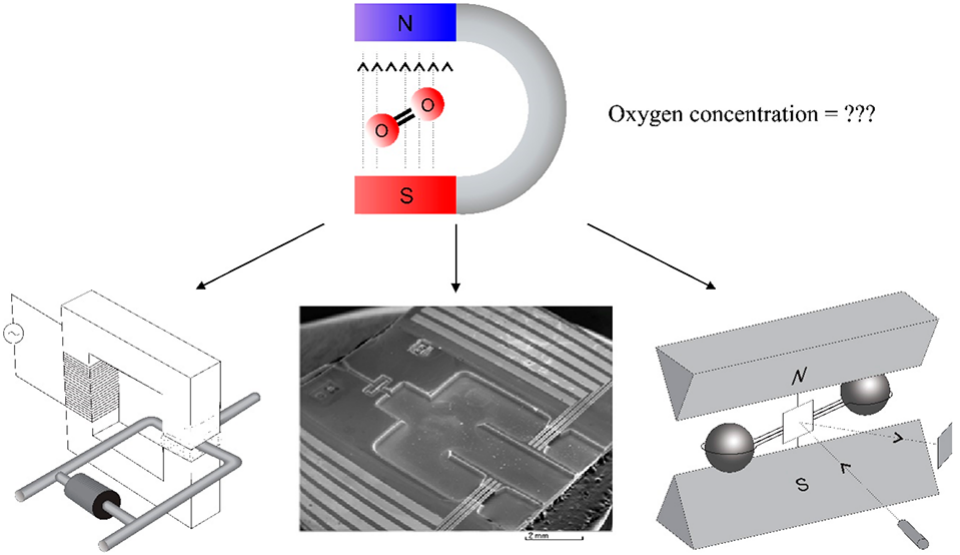

One of the most important methods of measuring the concentration of gaseous oxygen uses its paramagnetic properties, thanks to which oxygen molecules are drawn into the area of a strong magnetic field. This Review presents the current state of knowledge, achievements, and development prospects in the field of magnetic oxygen sensors using this phenomenon. We present the theoretical basis of the physical phenomena used in the paramagnetic oxygen sensors. The principles of operation of individual types of paramagnetic oxygen sensors, including the well-established and widely used magnetoacoustic and magnetopneumatic devices as well as the Pauling cells, are also described. In addition, this Review presents the existing and conceptual innovative sensors known mainly from the scientific and patent literature, including refractometric, interferometric, and ultrasonic sensors. This Review also discusses the advantages and limitations of individual devices, indicating the potential areas of their application.

The need to measure oxygen concentration occurs in many branches of industry, science, medicine, and everyday life. Areas in which the monitoring of oxygen concentration is of particular importance include the control of combustion processes (energy, automotive, chemical industry, etc.); the monitoring of metabolic processes (medicine, biology, agriculture, etc.); air quality monitoring in confined spaces or households; atmosphere monitoring in fruit storages, greenhouses, fermentation silos, and warehouses; the monitoring of potentially explosive areas; and in the cement industry. There are a number of analytical methods used to determine the gaseous oxygen concentration, the most important of which are electrochemical and magnetic methods.^1^ Electrochemical methods are based on the measurement of current or voltage during a chemical oxidation reaction. There are some distinct types of electroanalytical methods used for oxygen monitoring; the most important of them are polarography and electrocatalysis. The advantages of electrochemical sensors include the simplicity of their design, their high sensitivity, and their low unit cost. However, they possess a number of limitations related to the very strong temperature dependence of the signal, their short service life, the significant influence of interferents, and the possibility of poisoning by various chemicals. For this reason, magnetic rather than electrochemical methods are used in applications where high durability and accuracy of measurements are crucial (medicine, industrial process control, etc.). These methods take advantage of the fact that oxygen molecules are paramagnetic and are therefore drawn by the magnetic field into the area of greatest field strength.

A separate group of magnetic methods that can be used to determine the oxygen content are methods based on EPR (electron paramagnetic resonance) spectroscopy. These methods use the fact of splitting the energy levels of paramagnetic atoms in a magnetic field. EPR spectroscopy is commonly used to determine the structure of chemical compounds, and to study the mechanisms of chemical reactions and biochemical processes.^2,3^ However, it requires sophisticated measuring equipment and is mostly used for condensed-phase studies. The theory of EPR spectroscopy and the design solutions of EPR spectrometers constitute a separate, broad field of science and are not discussed in this work.

Paramagnetic oxygen analyzers are used in many fields of science, industry, and medicine, but the design of the sensors themselves is similar in all of these instruments. Often the same oxygen sensor is used in different instruments for many applications. The differences between oxygen analyzers usually come down to the method of sampling, gas mixture flow rate, measuring range, response time constant, or the method of visualization of measurement results. For this reason, this work focuses on the design solutions of the oxygen sensors themselves, giving examples of their use in commercial analyzers. Because of their common use, these are mainly analyzers used for fuel combustion control and respiratory monitoring in intensive care.

## Physical Basis of Paramagnetic Sensor Operation

Paramagnetic oxygen sensors use several physical mechanisms related to the effect of a magnetic field on oxygen molecules. These include the following: (1) the action of forces drawing oxygen into the area of the magnetic field; (2) changes in the magnetic susceptibility of oxygen as a function of temperature, resulting in a change in the magnetic forces acting on oxygen at different temperatures; (3) differences in paramagnetic gas pressures between areas with different magnetic field strengths; (4) the generation of gas flow or a change in its direction under the influence of a magnetic field; and (5) a change in other physicochemical properties of the fluid (density, refractive index, etc.). This chapter describes the most important physical relationships used in the design of paramagnetic oxygen sensors and presents them in a mathematically consistent form.

### Paramagnetism

**Figure 1:**
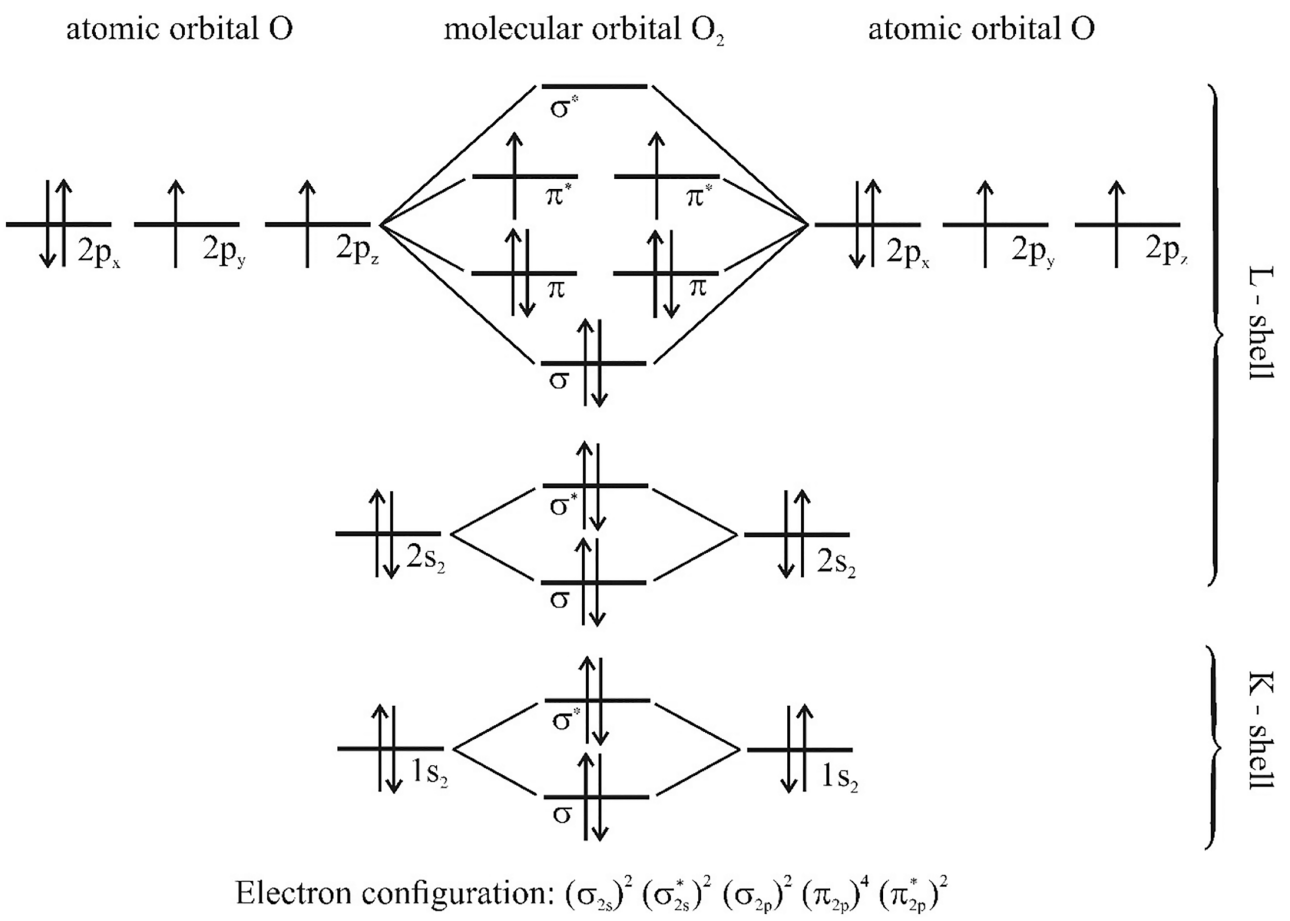
Molecular orbital diagram of O_2_. The paramagnetic properties of oxygen are due to the presence of two unpaired electrons in the L shell.^4^

Magnetism arises from the movement of electrons in atoms. Hund’s rule says that the molecular orbitals are occupied first by electrons with the same direction of magnetic moment (spin) and then by electrons with opposite spin. Orbitals that are completely filled with electrons do not exhibit magnetic properties. However, within orbitals with an unpaired electron, the magnetic effect is not balanced, and these electrons will order themselves according to the externally applied magnetic field. Figure 1 shows the electron configuration of the oxygen molecule.^4^

In the case of the two highest occupied orbitals (π*), the magnetic fields are not compensated; hence, there is a paramagnetic effect. O_2_ molecules strengthen the magnetic field, and in the external nonhomogeneous magnetic field they will be drawn into the area of greater field strength.

One of the most important properties of paramagnets is that, although their atoms have a constant, nonzero resultant magnetic moment, the interactions between these moments are very weak. Consequently, in the absence of an externally applied magnetic field, the resultant magnetization of the material is zero due to thermal fluctuations. Only when an external magnetic field is applied are the magnetic moments of individual atoms partially oriented, and the resultant magnetic moment appears toward the external field. Thus, the magnetic moment induced by the field is directed parallel to this field, and, hence, the paramagnetic susceptibility is positive, although it is a relatively small value.

Magnetic susceptibility is a measure of the paramagnetic properties of a medium. It is a dimensionless proportionality constant that represents the degree of magnetization of a material caused by an external magnetic field. The volumetric magnetic susceptibility (χ) is given by the following relationship:
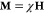
1where **M** [A/m] is the magnetization of the material (the magnetic dipole moment per unit volume) and **H** [A/m] is the magnetic field strength. The magnetic induction, **B**, is related to **H** by the following formula:
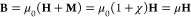
2where μ_0_ = 4π × 10^–7^ H/m is the vacuum permeability and μ = μ_0_(1 + χ) is the relative permeability of the material.

According to the Langevin theory,^5^ the following is given:
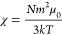
3where *N* is the number of atoms per unit volume, *m* is the permanent magnetic dipole moment of a molecule, *k* is Boltzmann’s constant, and *T* is the absolute temperature. Equation 3 results in Curie’s law, which has the following form:
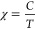
4where *C* is the material-specific Curie constant.

In addition to volumetric magnetic susceptibility, two other measures of magnetic susceptibility are distinguished, the mass magnetic susceptibility (χ_*m*_) and the molar magnetic susceptibility (χ_*M*_). Both quantities are defined by the following:
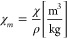
5
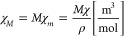
6where ρ is the density in kg/m^3^ and *M* is the molar mass in kg/mol. The above definitions (eqs 3–6) are according to the International System of Units (SI) conventions. However, many tables give values of magnetic susceptibility expressed in the centimeter-gram-second system of units (CGS). The dimensionless volumetric magnetic susceptibility value in the CGS system can be converted into the SI value by multiplying the CGS value by 4π.

The vast majority of gases that make up the air are diamagnets (nitrogen, argon, carbon dioxide, and water vapor), and they have a slight negative magnetic susceptibility. Among the components of air, only oxygen and nitrogen oxides (which may be present as air pollutants, but usually their content in the air is negligible) are paramagnetic. For this reason, the magnetic properties of oxygen can be successfully used for its determination both in air and in mixtures where other paramagnetic gases are not present in significant concentrations. The molar magnetic susceptibilities of the main components of air and its most common pollutants are presented in Table 1.

**Table 1:** Molar Magnetic Susceptibility of the Components of Air and Typical Pollutants at Room Temperature^6^

	gas								
	O_2_	N_2_	H_2_O vapor	Ar	CO_2_	CO	NO	NO_2_	SO_2_
χ_M_ × 10^10^[m^3^/mol]	+429.1	–1.51	–1.65 (373 K)	–2.43	–2.64	–1.48	183.6	+18.8 (408 K)	–2.29

### Magnetic Forces and Their Influence on the Flow of Paramagnetic Fluids

Considering the force, **F**_***m***_, acting on the dipole in an externally applied magnetic field, **H**, the Kelvin dependence is obtained by the following:^7^
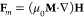
7

By treating the medium as a set of dipoles, this formula can be used to determine the magnetic force acting on a single element of the medium. This dependence does not take into account the modification of the applied magnetic field by the dipoles of the medium, but in the case of para- and diamagnetic media, the changes in the external field are negligible.

For paramagnetic fluids, after substituting formulas 1 and 5, relation 7 takes the following form:
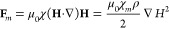
8

As a mixture of gases, air has a volumetric susceptibility of the following:
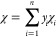
9where *y*_*i*_ is the volume fraction of the substance *i* in air and χ_*i*_ is the volumetric magnetic susceptibility of the substance *i*. Because the volumetric susceptibility of oxygen is much greater than that for other air components, the formula for the magnetic susceptibility of air is simplified to the following:
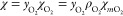
10where the O_2_ index denotes gaseous oxygen.

Using the relationship for the strength of magnetic field:
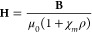
11the magnetic mass force can be approximated by the following relationship assuming that χ_*m*_ρ ≪ 1:
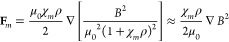
12

By comparing the molar magnetic susceptibility of the main components of air, measured at room temperature (Table 1), it can be seen that this assumption is fully justified.

The Kelvin force (eq 12) can be introduced into the Navier–Stokes momentum equation as the external force acting on the differential element of the fluid,^8^ as follows:
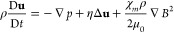
13where D**u**/D*t* is the material derivative of flow velocity vector **u** with respect to time *t*, *p* is the pressure, and η is the fluid viscosity.

If χ_*m*_ and ρ are constant and there are no forces acting on the fluid other than the magnetic force, it remains at rest (**u** = 0), and eq 13 takes the form:
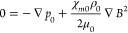
14where subscript 0 in *p*_0_, χ_*m*0_, and ρ_0_ indicates the static state.

If there is a temperature difference in the medium, the magnetic permeability and density of the fluid have different values depending on the local temperature. The pressure, *p*, then can be expressed as the sum of the static pressures, *p*_0_, and its disturbance, *p*′:
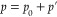
15

By subtracting eq 14 from eq 13, we obtain the following:
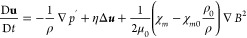
16

For slight temperature differences, the magnetic susceptibility can be expanded in the Taylor series of (*T* – *T*_0_) as
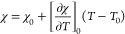
17where, according to eq 4:
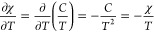
18

The gas density can by approximated as follows:
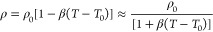
19

Hence, the momentum eq 16 takes the following form:
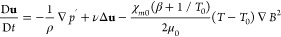
20

This formula shows that the magnetic buoyancy term is dependent on the gradient of the square of the magnetic induction and the temperature difference.

### Pressure Difference

The basis of the operation of many paramagnetic oxygen analyzers is to create a pressure difference between regions of different magnetic field strength.

**Figure 2:**
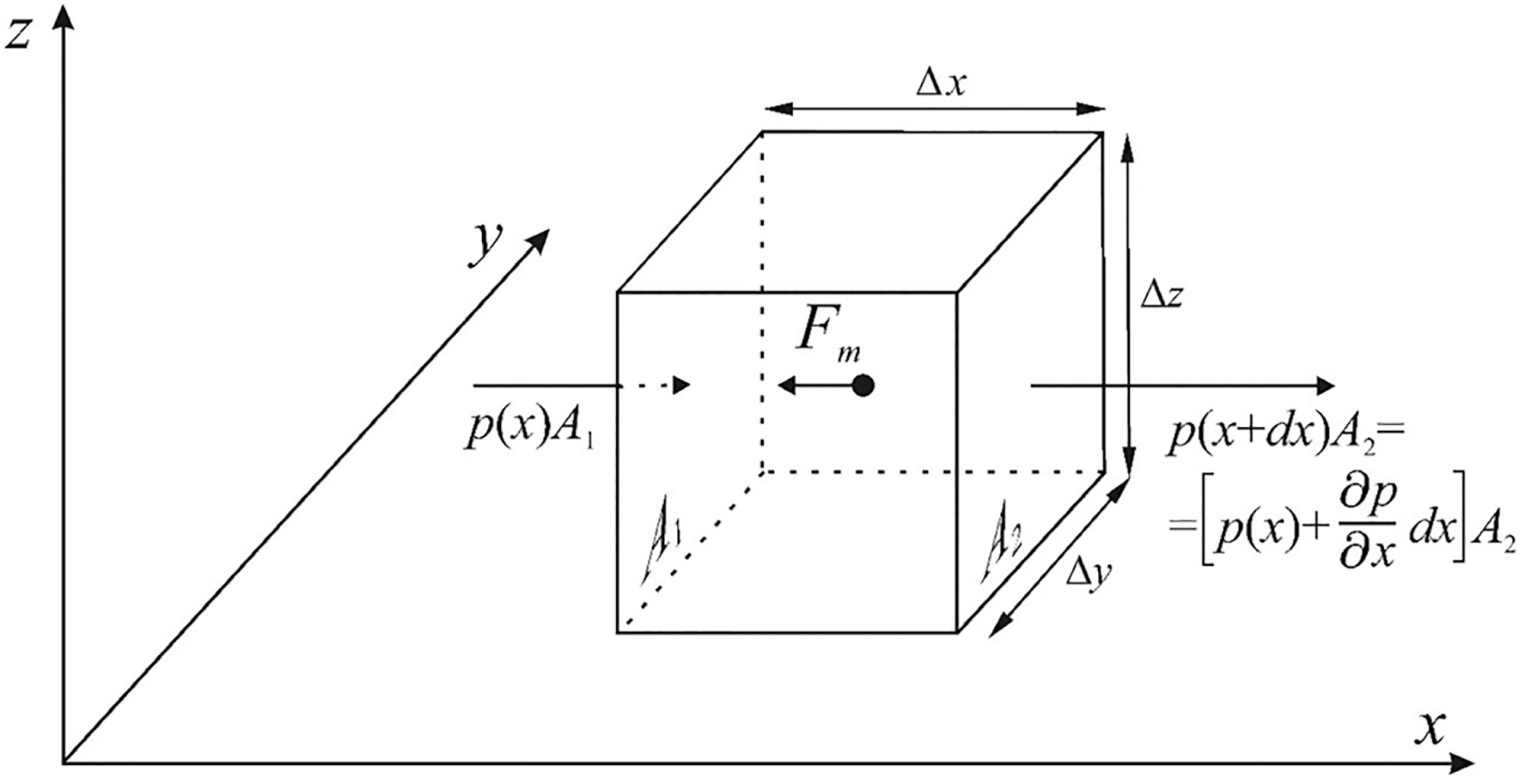
Element of gas volume d*V* in the nonuniform magnetic field.

To determine the value of the pressure difference, let us consider the elementary volume of gas d*V* = d*x* d*y* d*z*, placed in an inhomogeneous magnetic field of intensity **H** (Figure 2). According to the ideal gas law, the number of moles of oxygen in the volume d*V* is equal to
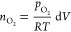
21where *p*_O2_ is the oxygen partial pressure and *R* is the gas constant.

Let us perform a balance of forces acting on this d*V* element along the *x*-axis. The pressures *p*(*x*) and *p*(*x* + d*x*) = *p*(*x*) + ∂*p*/∂*x* d*x* are exerted on the surfaces *A*_1_ and *A*_2_, respectively. Hence, the force resulting from the pressure difference is equal to
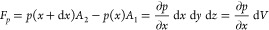
22

At equilibrium, this force is counterbalanced by the magnetic force:
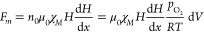
23

By comparing eq 21 and eq 22, we get the following differential equation:
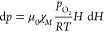
24

This equation can be integrated in the range from *p*_0_, where there is no magnetic field (*H* = 0), to the *p*, where the value of the magnetic field intensity is *H*_0_:
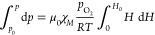
25

Hence:
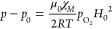
26

It should be emphasized that the pressure difference is independent of the spatial distribution of the field between 0 and *H*_0_.

Formula 26 allows for estimation of the pressure increase in the magnetic field for typical operating conditions of the oxygen sensor. Assuming *B* = 1 T, with a pressure of 1 atm and a temperature of 298 K, the increase in oxygen pressure is 0.70 Pa. In turn, an increase of 100 K in temperature under these conditions causes a change in oxygen pressure of −0.18 Pa.

## Technical Solutions of Paramagnetic Oxygen Sensors

There are different criteria for the classification of paramagnetic oxygen sensors. The most general division takes into account the type of magnetic field used. Devices equipped with permanent magnets are called magnetostatic, while devices equipped with electromagnets generating an alternating magnetic field are called magnetodynamic.

Sintered rare earth oxide magnets with a volume of several milliliters can create a magnetic field of up to 1 T; therefore, magnetostatic sensors can be small and have low power consumption. Usually, measurements made with such instruments are slow. Time constants range from a few seconds to several seconds. However, this is not so much due to their operating principle as to their design solutions because their correct operation requires a small and stable flow of the measured gas. Significant increases in the measurement speed are observed in microelectromechanical system (MEMS) designs, where the miniature size and small dead volumes ensure rapid gas exchange even at very low flow rates.

Magnetodynamic oxygen sensors use a strong, fast-changing electromagnetic field in the air-gap of electromagnets, in which oxygen molecules vibrate at a frequency corresponding to the frequency of the current driving the coil. The intensity of particle vibrations can be measured with a microphone or other sensor. Because of the energy losses in the core of the electromagnet, the typical operating frequency corresponds to the lower range of acoustic frequencies between 100 and 200 Hz. Because of the principle of operation, these sensors are also called magnetoacoustic. The great advantage of magnetoacoustic oxygen sensors is their very short response time. The disadvantage, however, is the large size resulting from the presence of the electromagnet and the high power consumption. These types of sensors are now widely used in intensive care monitors. Measurement with magnetoacoustic sensors requires a continuous flow of the gas mixture through the measuring chamber. For their operation, a reference gas is required, which must be mixed with the measured gas within the area of the homogeneous magnetic field. In most applications, ambient air can be used as a reference gas, and the differential principle of operation is especially useful in cases where the difference in oxygen concentration of two different gases needs to be determined. In practice, the need to use a reference gas necessitates a more complex structure, larger dimensions, and the use of pneumatic balance. In addition, the microphone used must be characterized by high sensitivity, stability, and tightness.

Both magnetostatic and magnetodynamic devices use various physical phenomena occurring under the influence of a magnetic field in a gas mixture containing paramagnetic oxygen. There are practically no oxygen sensors that measure the absolute values of the physical parameters of the gas, that is, pressure, magnetic susceptibility, viscosity, or refractive index. This is due to the difficulty of measuring very small changes in the signal against the large DC component. Therefore, differential measurements are made with respect to a gas not subjected to a magnetic field, or the magnetic forces acting on the gas are measured because in the absence of oxygen they are equal to zero.

The physical phenomena most commonly used to measure oxygen concentration are as follows: (1) the buoyancy force acting on a diamagnetic gas in a closed vessel surrounded by a paramagnetic gas, placed in a constant magnetic field (Pauling cell); (2) the periodic changes in pressure or flow in the pneumatic bridge, which arise as a result of applying an alternating magnetic field to one of the bridge arms (magnetoacoustic and magnetopneumatic sensors, respectively) (these systems require the use of a reference gas, usually air); (3) “thermo magnetic wind” generated by differences in the magnetic susceptibility of different regions of the gas, which are the result of its unequal heating (in a constant magnetic field, areas of gas with greater susceptibility are then subjected to greater force); (4) the deviation of the gas flow direction from a straight line under the influence of a constant magnetic field; (5) changes in magnetic field strength induced by paramagnetic oxygen; and (6) changes in other physicochemical properties of paramagnetic gas, such as the refractive index.

The following sections present the design solutions of sensors using individual types of the physical phenomena to measure oxygen concentration and new concepts appearing in the scientific and patent literature.

### Sensors Measuring Buoyancy Force

**Figure 3:**
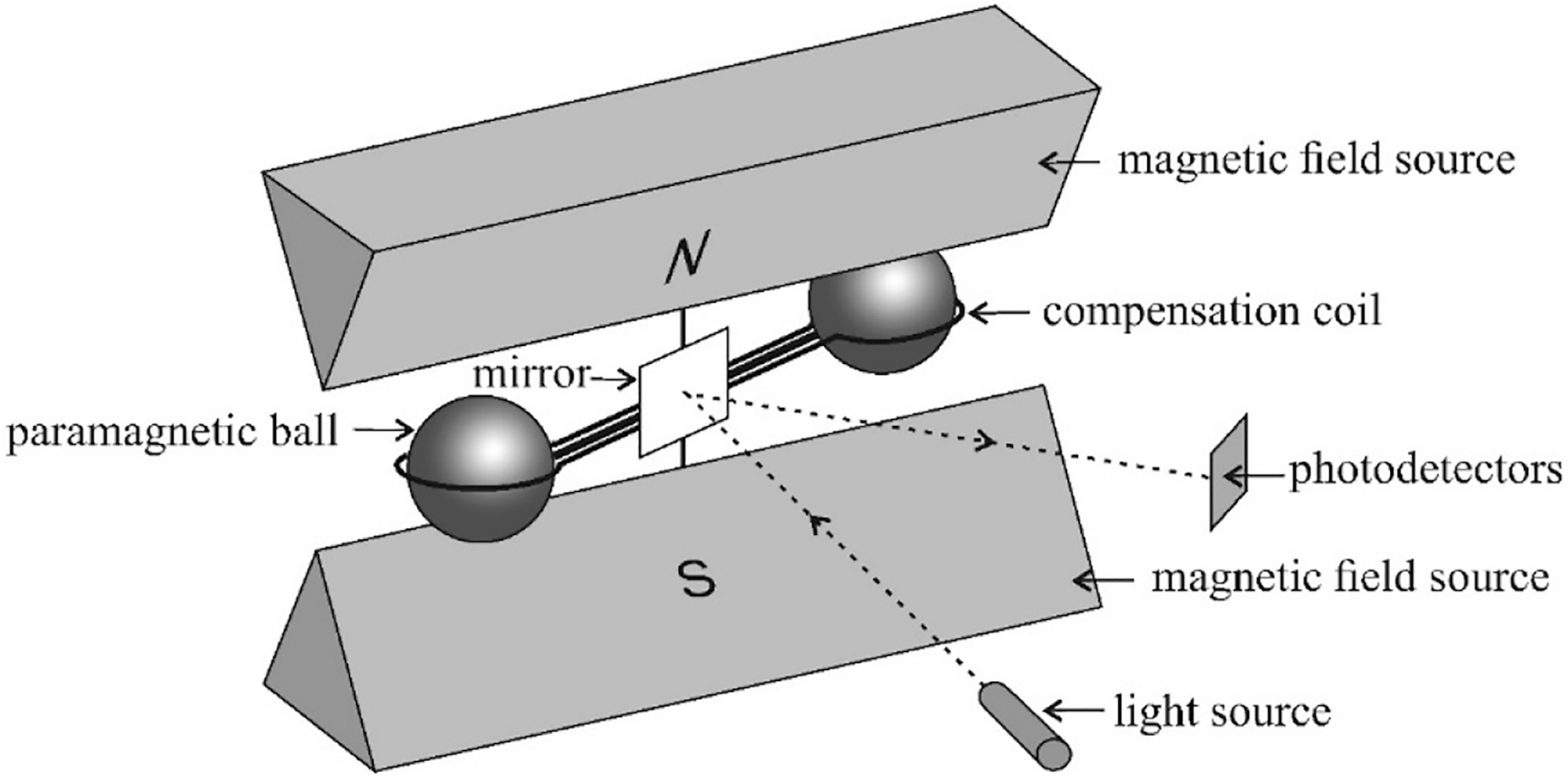
Schematic diagram of the Pauling cell.^12^

**Figure 4:**
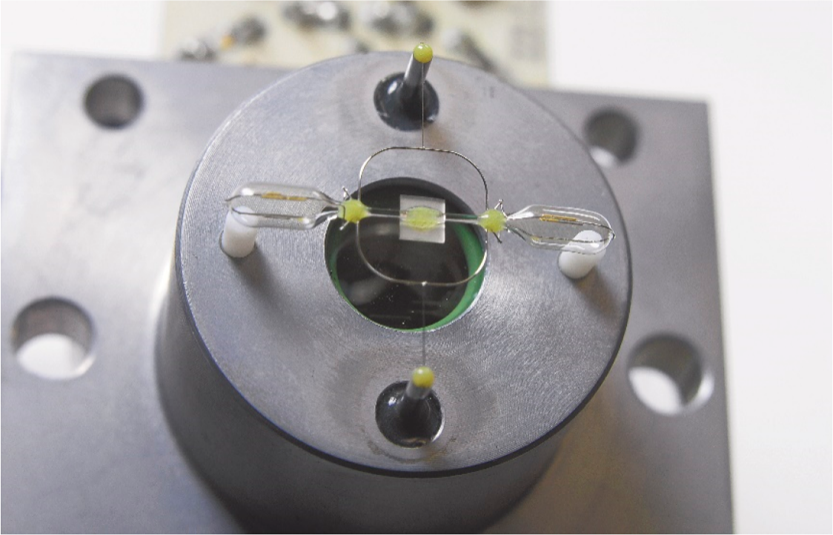
Photo of the Pauling cell from an ABB Ltd. analyzer. Reprinted with permission from ref (13). Copyright 2006 ABB Review.

The first designs of devices measuring magnetic buoyancy force were developed in the late 1930s and early 1940s.^9^ The oldest solution of this type is the Pauling cell. In this type of sensor, two nitrogen-filled glass spheres are connected to each other like dumbbells and mounted on a rotating suspension (quartz thread). This assembly is placed in the field of a strong permanent magnet (Figures 3 and 4). A stream of the analyzed gas mixture (several milliliters per minute) flows around it. Because of the paramagnetic properties of oxygen, its molecules tend to accumulate between the poles of the magnet, what increases the gas pressure locally. As a result, nonparamagnetic dumbbells are pulled out of the equilibrium position. The movement of the assembly is optically detected. A small mirror attached to a quartz thread (and rotating with the assembly) reflects the light beam on a scale calibrated in oxygen concentration or partial pressure units. There is a drying agent at the inlet to the chamber, which is silica gel. A description of the design and application of the direct reading analyzer can be found in work by Woolmer.^10^

To obtain greater accuracy, modifications are made to the Pauling cell enabling measurement by the null balance method. The deflection of the light spot in the analyzer is compensated by a current flowing in a coil surrounding a quartz dumbbell pendant. A multiturn potentiometer, which controls the compensation current, is calibrated in oxygen concentration units. An example of such an analyzer is DLC.101 produced by Servomex Controls Ltd.^11^ It has a measuring range of 0–100% oxygen and a potentiometer graduated in 0.1% steps. The device provides an accuracy of ±0.1% for a 5 K change in ambient temperature. The operating temperature range is 263–313 K, and the optimal sample flow rate is 100 mL/min. The tilt is exponential, and a 90% response time is less than 8 s.

The contemporary design of the Pauling cell, used in ABB Advance Optima and EasyLine^12,13^ oxygen analyzers, is shown in Figure 4. The cell has an internal volume of approximately 6 mm^3^, and the diameter of the glass probe body is 2 mm. Such classic paramagnetic sensors have a number of significant advantages. They have a short response time (3–10 s) limited mainly by the gas exchange rate in the relatively large volume of the sensor. Interferences with diamagnetic gases are negligibly low. Currently, most of the commercial devices using oxygen paramagnetism are dumbbell constructions. They are produced by, among other companies, ABB,^14^ Servomex,^15^ Ankersmid,^16^ Teledyne Analytical Instruments,^17^ Sigas,^18^ LFE Process Analytical Instrumentation,^19^ Systech Illinois,^20^ and Fuji Electric.^21^ The improvement of this classic design is also ongoing, in terms of both mechanics and signal processing, as reflected in the patent literature.^22−26^

Sensors of this type allow for a very precise and accurate measurement of the oxygen content in gas mixtures. High-quality dumbbell sensors can achieve a resolution and accuracy better than 100 ppm in the oxygen concentration range of 10–100%.^27^

In recent years, developments in materials and electronics engineering have led to miniaturization and further significant improvements in cell performance, while maintaining the original operating principle. A response time of 1 s has been achieved, which extends the range of applications of this sensor to fast-changing processes. In addition, the degree of complexity has been reduced, which shortened the production time of the devices. An example is the MEMS sensor, described in the literature,^12^ which consists of the same functional elements as the classic structure shown in Figure 4.

**Figure 5:**
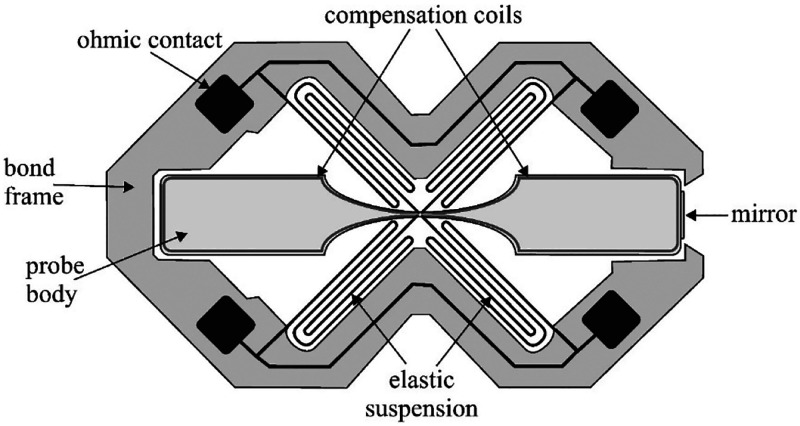
MEMS sensor for the paramagnetic oxygen measurements.^12^

The sensor has a rotating body probe in the shape of a paddle with dimensions of 3 × 7 mm, and it is suspended on folded flat springs (Figure 5). The compensation coil is structured on the edge of the body probe. On the spring surface, there are conductive tracks connecting the compensation coil with the pads (ohmic contacts) on the bond frame. The gas channels and the inlet to the sensor have been designed in such a way as to minimize the gas exchange time in the sensor, at the same time not causing signal disturbances that may occur with a fast gas flow. As in classic solutions, the position of the probe is determined optically through a window located in the sensor housing.

The above sensor has a detection limit of 50 ppm of O_2_ at 1 Hz and a response time of 1.3 s. The influence of the flow rate was determined as ±70 ppm of O_2_ in the flow range of 6–7 mL/min.

The obtained results show that this sensor has appropriate parameters for a number of industrial applications; however, similar to classic solutions, the sensor is sensitive to vibrations, and, therefore, its use is limited to stationary applications.

**Figure 6:**
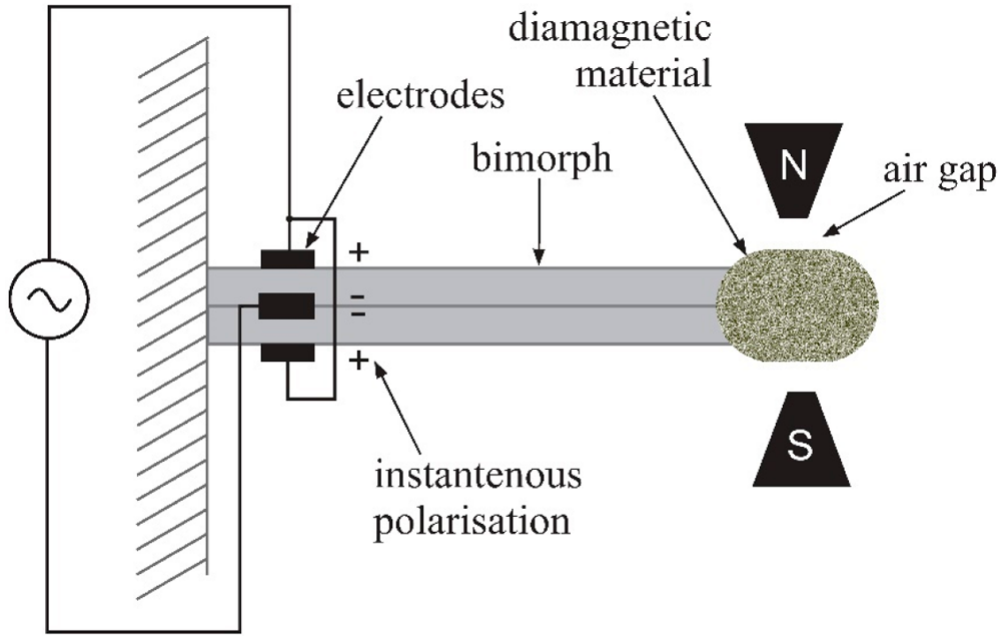
Oxygen sensor with a bimorphic resonator.^28^

Another sensor design based on the force measurement exerted on a nonmagnetic body placed in a magnetic field is described in other work.^28^ This is achieved by the use of a piezoelectric bimorph. It is composed of two piezoelectric plates, placed one of the top of other, attached with one end to a wall of casing (Figure 6). The second end is inserted into a nonmagnetic body and placed in a magnetic field between poles of a permanent magnet. An alternate voltage induces deflection of the bimorph, generating a pressure variation. This pressure variation induces a variable load, which may be processed by dedicated electronics. The signal frequency is matched to the mechanic resonance of the transducer.

Experimental studies have shown that the response time for oxygen concentration in the range of 10–90% is equal to 95 ms, and the concentration determination error is less than 3%.^28^

### Magnetoacoustic and Magnetopneumatic Sensors

**Figure 7:**
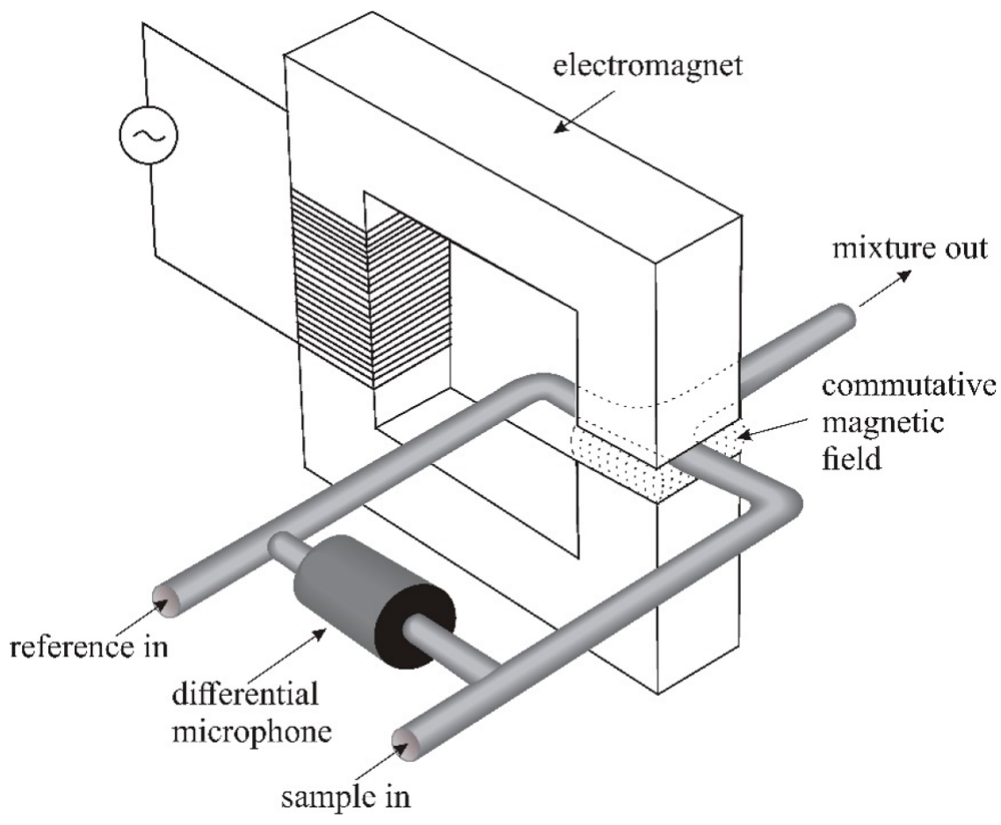
Design of a magnetoacoustic sensor.^30^

**Figure 8:**
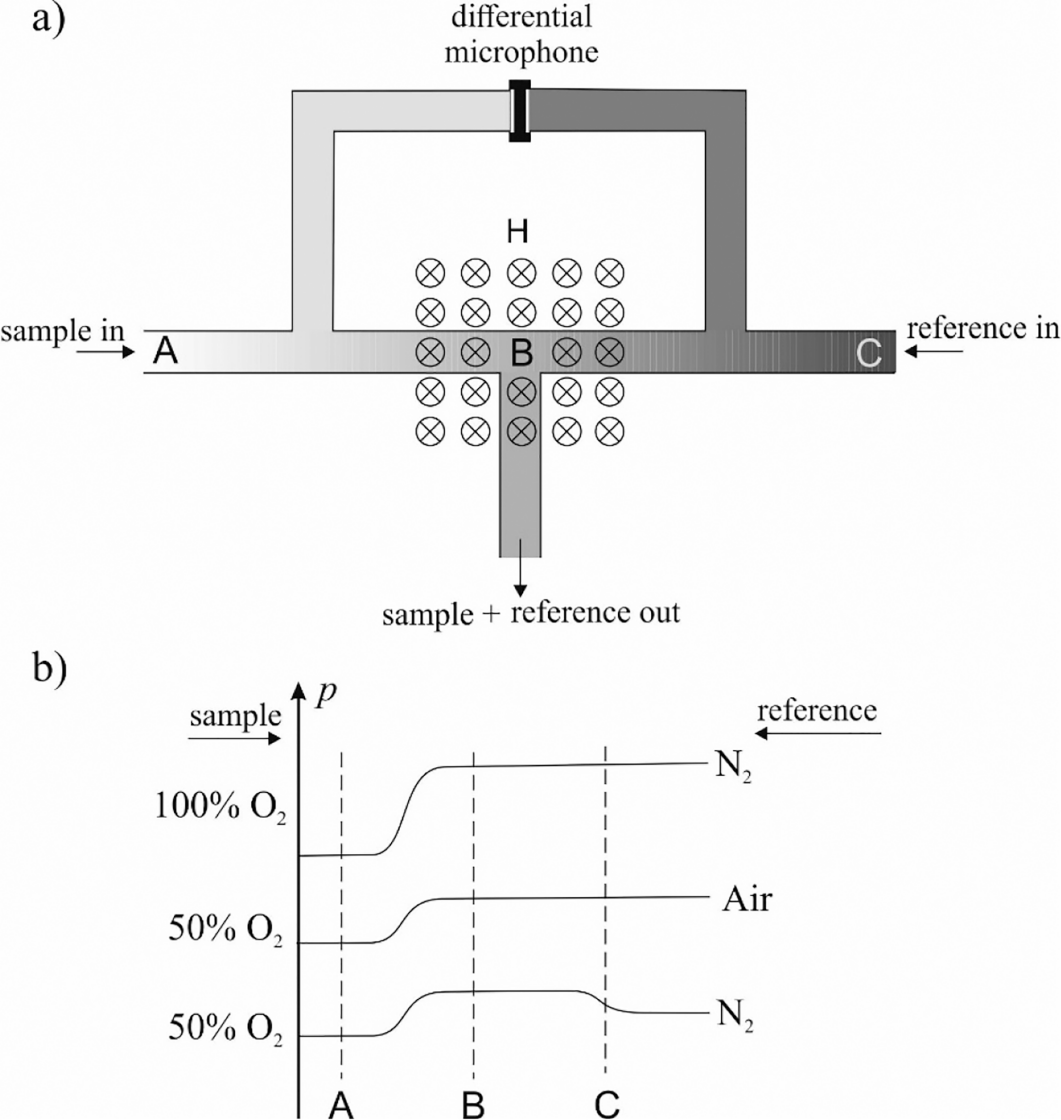
(a) Scheme of the measuring cell. The symbol ⊗ indicates the magnetic field perpendicular to the drawing plane. (b) Pressure distributions inside the ABC gas lines for different oxygen contents in the sample and the reference gas.

The first magnetoacoustic sensor was developed by Hummel.^29^ He built a cell in which two gases mix in a homogeneous magnetic field (Figure 7). In addition to the gas to be measured, a reference gas, usually air or nitrogen, is fed into the gap. The alternating magnetic field causes periodic pressure changes in the gap and gas lines to which the differential microphone is connected (Figure 8).

Figure 8 shows the operation diagram of the magnetoacoustic sensor and the pressure distributions in the gas lines for different oxygen contents in the sample and in the reference gas. The pressure measured by the microphone corresponds to the pressure difference at inlets A and C. Apart from the pressure drop due to flow resistance, the pressure at point B, where the sample and reference gas lines connect, is the same for both gases. However, along the AB and BC lines, an increase in pressure occurs proportional to the partial pressure of oxygen. It follows that if the oxygen content in both is different, the pressure difference will be proportional to the oxygen concentration difference when the magnetic field is turned on. Hence, the amplitude of the output signal at a constant amplitude of an alternating magnetic field and a constant temperature is given by the following formula:
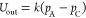
27where *k* is a constant depending on the design of the microphone.

The small volume of the measuring cell ensures a short response time, which makes the system suitable for medical applications, for example, in anesthesia. Using an appropriate design, noise levels of less than 0.03% oxygen can be achieved. Magnetoacoustic sensors were used in metabolic and respiratory care monitors manufactured in the 1970s and 1980s by Godard,^31^ Hartmann and Braum,^32^ and by Datex Instrumentarium Corp.^30,33^ This principle of operation is also used in analyzers for combustion control and industrial processes.^34^ Although magnetoacoustic instruments were among the first to be used to measure oxygen concentration, this technique is constantly being improved.^35−37^

The analyzer containing the microphone is inherently sensitive to mechanical vibrations and sounds; therefore, it is important that the design isolates the microphone from the environment as much as possible.

The differential measuring principle as used in magnetoacoustic sensors is an advantage in applications such as measuring oxygen consumption. However, the need for a continuous reference gas supply is a significant disadvantage in closed anesthetic circuits where air cannot be used as a reference gas because this would result in a slow accumulation of nitrogen in the respiratory system. Therefore, other designs of magnetoacoustic sensors are being developed, in which the acoustic wave generated directly in the electromagnet gap is measured. The alternating magnetic field causes the oxygen molecules within the electromagnet gap to vibrate synchronously with the current fed to its winding. The resulting acoustic wave is detected by a microphone located near the aperture. Such a system does not require the use of a reference gas and a complex gas system; however, so far, descriptions of such systems can be found mainly in patent applications.^38−40^ Therefore, there is no reliable information on their metrological parameters.

The structure of magnetoacoustic sensors is similar to that of magnetopneumatic sensors. The only difference in design is that instead of a microphone measuring the pressure difference in the pneumatic bridge, microflowmeters are used to measure the flow between the bridge arms. The advantage of this type of construction is the elimination of a microphone that is sensitive to vibrations and temperature changes. The differential operation of thermoanemometric flowmeters also ensures very good compensation of temperature and power fluctuations. The disadvantage is that the signal is smaller and the time constant is many times greater than that of the microphone. This limits the frequency of the magnetic field modulation to several Hz, as compared to 100–200 Hz in devices with a microphone.

Magnetopneumatic sensors are widely used in modern designs of oxygen analyzers. Examples are instruments from Fuji Electric,^41^ Siemens,^42^ and Horiba,^43^ which provide a measurement error of less than 1% O_2_ for a measuring range of 0–100% O_2_, with a time constant of 1.5–3 s.

### Magnetic Wind Oxygen Sensors

**Figure 9:**
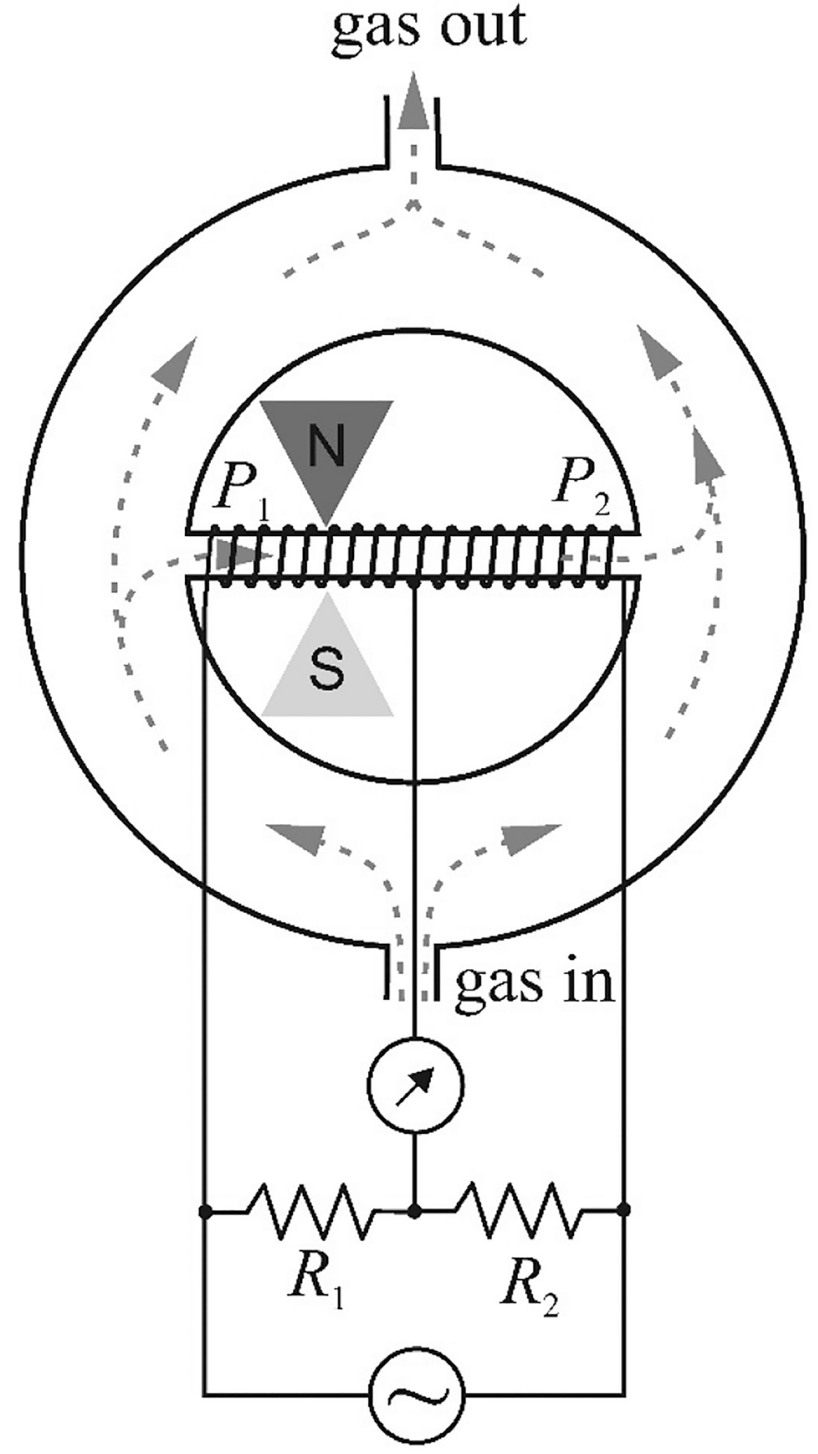
A diagram of the operation of one of the types of devices using magnetic wind.^47^

The movement of paramagnetic oxygen molecules toward the magnetic field is called a magnetic wind. A detailed description of the early use of this phenomenon can be found in the literature.^44−46^ A schematic diagram of such an analyzer is shown in Figure 9.

The inlet gas stream flows through a ring-shaped measuring chamber. A thin-walled glass tube passes through the center of the ring, providing a direct gas connection between the left and right sides of the measuring chamber. A heating wire is wound around the glass tube to form the two arms, P1 and P2, of the AC bridge. The left part of the tube (at the point indicated in Figure 9) is placed between the poles of a permanent magnet in such a way that the magnetic field lines are perpendicular to the plane of the drawing.

If there are regions with different temperatures, *T*_1_ and *T*_2_, in a homogeneous magnetic field, the force, *F*, attracting the cold element of the oxygen volume is proportional to the following:
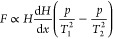
28where *H* is the magnetic field strength, d*H*/d*x* is the field gradient, and *p* is the oxygen partial pressure. Therefore, into the glass tube, cold oxygen-containing gas is drawn from the left side. Once the gas is drawn into this part of the tube, it is heated and loses its magnetization. It is then pushed out by the cold gas entering the tube from the left side, which creates a flow of gas (so-called magnetic wind) through the tube. Furthermore, the flow cools the P1 winding in relation to P2 and causes an imbalance of the AC bridge. The resulting temperature difference between the arms is influenced by the specific heat of the gas and its flow rate through the tube. The bridge signal, therefore, depends not only on the oxygen concentration but also on the specific heat and the viscosity of the gas mixture. In turn, the analyzer output voltage, *V*, depends on many factors, including the bridge current, magnetic field strength of the permanent magnet, ambient temperature, absolute gas pressure, type of carrier gas, and oxygen concentration. In fact, the output voltage usually decreases by about 1.5% per 1 K change in temperature and increases by 1.8% with a pressure increase of 1 kPa.^47^ Therefore, the analyzers are usually thermostated and often pressure compensated. The bridge output is nearly linear up to 10% O_2_. The zero settings depend on, among others, the position of the tube due to the influence of natural convection.

**Figure 10:**
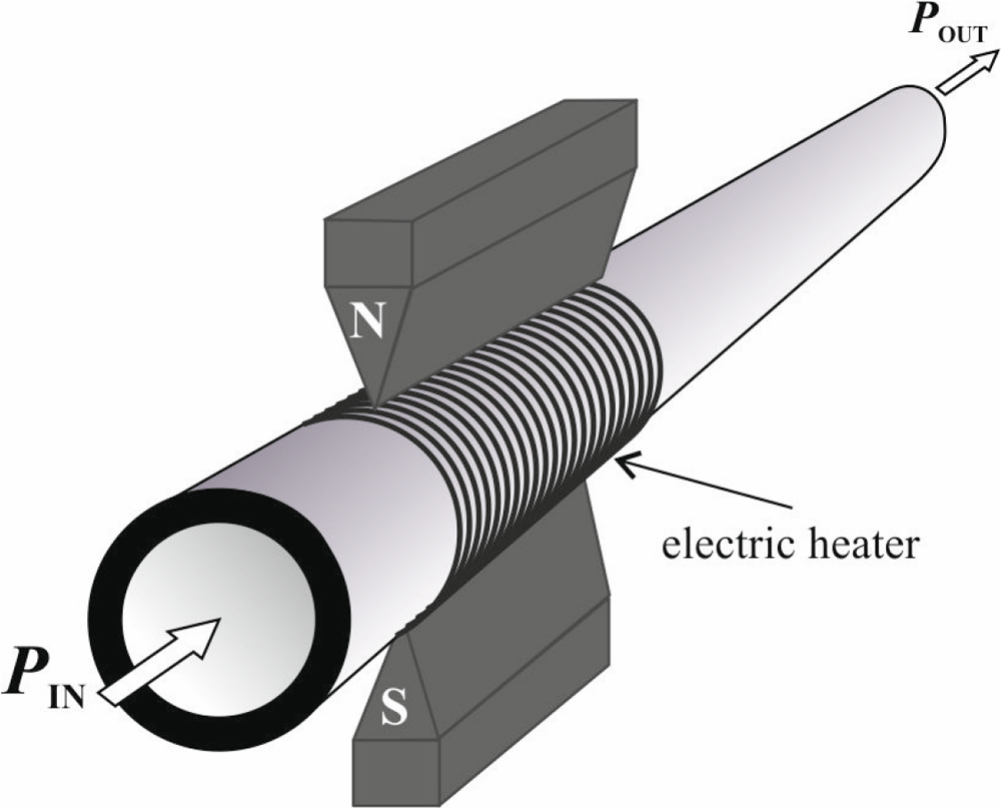
Schematic view of a thermal magnetic wind oxygen sensor.^48^

Numerical analysis of the magnetic wind phenomenon in a cylindrical pipe is presented in ref (48). The study considered a pipe with a diameter of 6 mm and a length of 28 mm with a heated zone (10 mm long) placed in a constant magnetic field (Figure 10).

The study assumes a steady state, laminar gas flow through the pipe and that the gas is incompressible. Moreover, it was assumed that with the temperature change, only the magnetic susceptibility of the gas changes and the other physical parameters remain unchanged. The effect of natural convection was also ignored. Magnetic thermal convection has been found to significantly increase the average velocity of gas flow through the pipe and heat transfer coefficient. For 1.32 T magnetic induction, 200 W/m^2^ heat flux, and 0.008 Pa in inlet and outlet pressure difference, when the oxygen concentration in the gas changes from zero to 100%, the average gas flow velocity increases by 70.7%, and the temperature of the tube wall changes by 15 °C. As the pressure difference between the inlet and outlet increases, the thermal-magnetic convection weakens. Only when the pressure difference is less than 0.014 Pa can the influence of thermal-magnetic convection be observed. The resolution of the tested system was estimated at approximately 0.0067% of oxygen concentration.

In other work,^49^ one can find theoretical considerations on the possibility of miniaturization of this type of sensor and its implementation in low-temperature cofired ceramics (LTCC) technology.

**Figure 11:**
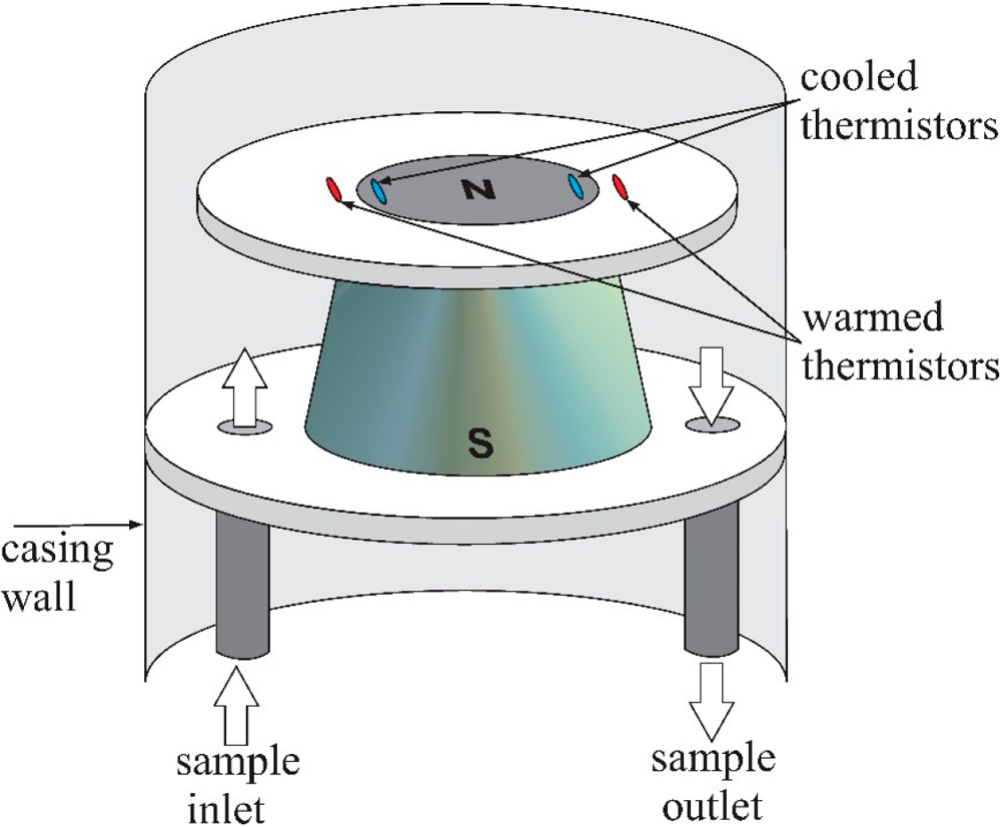
Structure and principle of operation of the XMO2 sensor from General Electric.^50^

A contemporary instrument using the magnetic wind method is the XMO2 analyzer from General Electric.^50^Figure 11 shows the structure of the used sensor and its principle of operation.

**Figure 12:**
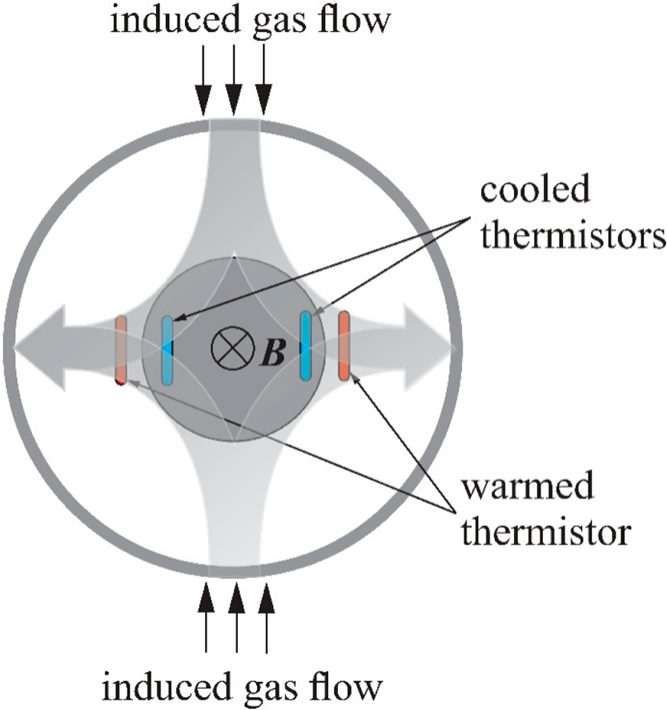
Arrangement of the thermistors in the XMO2 sensor.^50^

The sensor contains a permanent magnet located in the center of the cell. Two pairs of thermistors are placed above one of its poles in such a way that one thermistor of each pair is in the strong magnetic field and the other is beyond it. The thermistors are electrically heated, and the entire cell is thermostated to 45 °C. Figure 12 shows the arrangement of both pairs of thermistors.

A small portion of the measured gas diffuses from the lower to the upper part of the measuring chamber. The presence of paramagnetic gas causes an increase in pressure in the center of the chamber, where the magnetic field strength is greatest. At the same time, the pressure of the gas to be measured is somewhat lower near the thermistors because their high temperature reduces the magnetic susceptibility of oxygen. This slight pressure difference causes gas to flow from the center of the magnetic field outward over the thermistors. As a result, the internal thermistors cool, while the external thermistors, influenced by the warm gas, heat. Both pairs of thermistors are placed in the arms of an electric bridge that measures the asymmetry in the resistance induced by the temperature difference of the thermistors. A signal from the bridge is proportional to the oxygen concentration in the measured gas. This sensor has an accuracy of 1% in the range of 1–100% oxygen, with a linearity of ±0.5% of the measuring range and a response time of not more than 5 s. The influence of flow rate in the range of 50–1000 mL/min is less than 1% of the scale. The effect of pressure is ±1.5% per kPa (without compensation).^50^

### Sensors with Deflection of the Gas Stream

**Figure 13:**
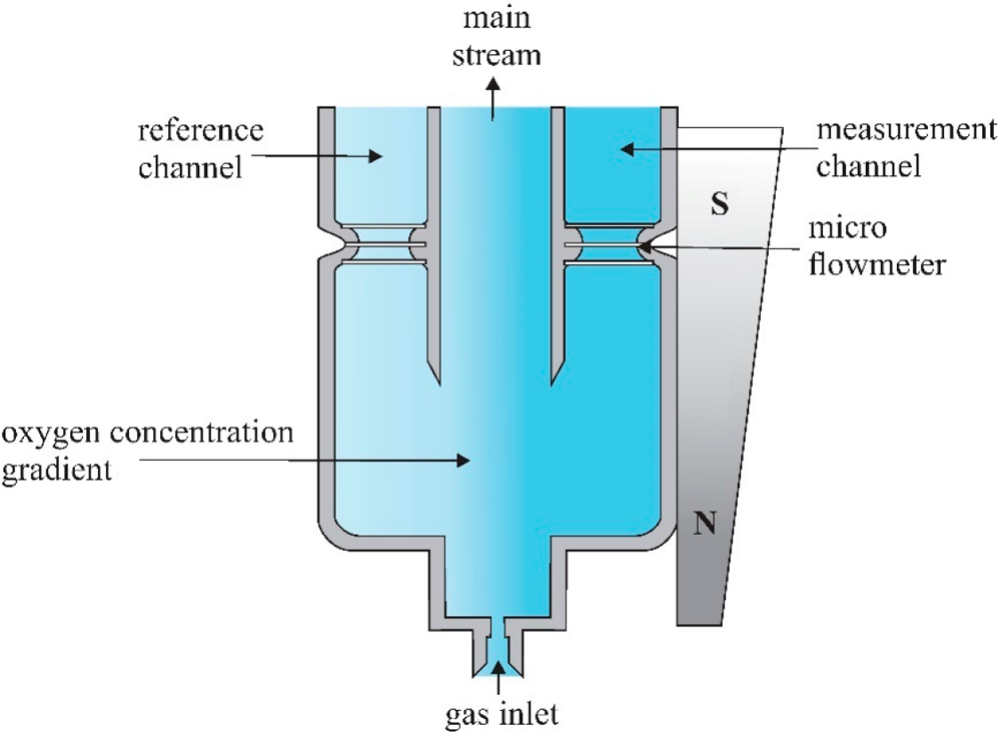
Diagram of the construction and operation of the sensors with deflection of the gas stream.^4^

Another method of measuring oxygen involves changing the direction of its flow in a magnetic field. This causes a partial separation of oxygen from the remaining components of the gas mixture. An example of such a sensor was described in the literature.^4,51^ The schematic diagram of this sensor is shown in Figure 13. Behind the gas inlet, there is an area where the stream of oxygen molecules is deflected in a nonuniform magnetic field generated by a suitably shaped permanent magnet placed on the side of the sensor. The gas stream is distributed into three channels: reference, central (main), and measurement channels. Thermoanemometers in the reference and measuring channels measure the gas flow velocity.

The measured gas introduced into the sensor is subjected to an inhomogeneous magnetic field. The diamagnetic gas interacts very weakly with the magnetic field and flows mainly through the central channel. In the measurement and reference channels, the flow velocity assumes the minimum value. Because of the symmetry of the sensor, the flows in the reference and measurement channels are the same.

If the gas contains paramagnetic oxygen, it is deflected toward the measuring channel, increasing the flow velocity in it and, at the same time, reducing the flow velocity in the reference channel. The change in flow velocity depends on the oxygen concentration in the measured gas. Thermoanemometers incorporated into the Wheatstone bridge measure the difference in gas flow velocity between the measuring and reference channels, which allows for the determination of the oxygen concentration in the measured gas.

The main advantages of this type of sensor result from the division of the measured gas stream and the side arrangement of the permanent magnet. Because of the geometry of the sensor channels, the diamagnetic gas flows mainly through the central channel, which results in a low gas flow velocity through the reference and measurement channels. Low flow velocities make it possible to accurately measure very small flow changes. The operation of thermoanemometers in a bridge system enables compensation of the influence of temperature and pressure.

The lateral position of the permanent magnet makes it possible to increase its size, unlike other sensors operating on the principle of changing the flow velocity, where the size of the magnet is limited by the size of the channel in which the measured gas is subjected to the magnetic field. By selecting a larger, stronger magnet, the accuracy of the sensor increases.

**Figure 14:**
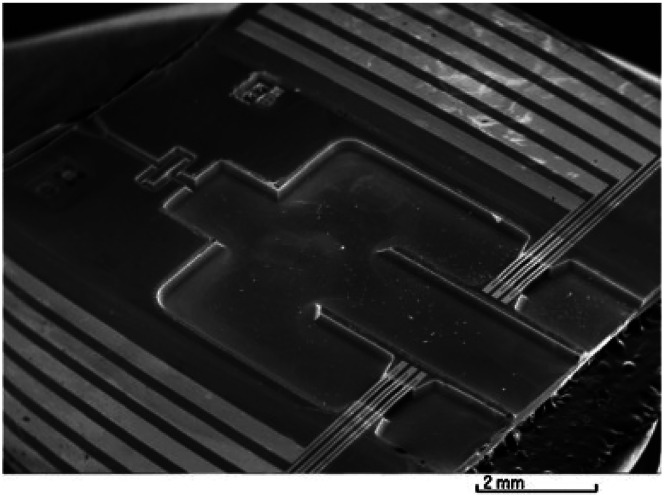
SEM image of a flow separation sensor. Reprinted with permission from ref (4). Copyright 2013 Elsevier.

The sensor described in previous work^4,51^ was fabricated using MEMS technology. The scanning electron microscope (SEM) picture of this sensor is shown in Figure 14.

**Figure 15:**
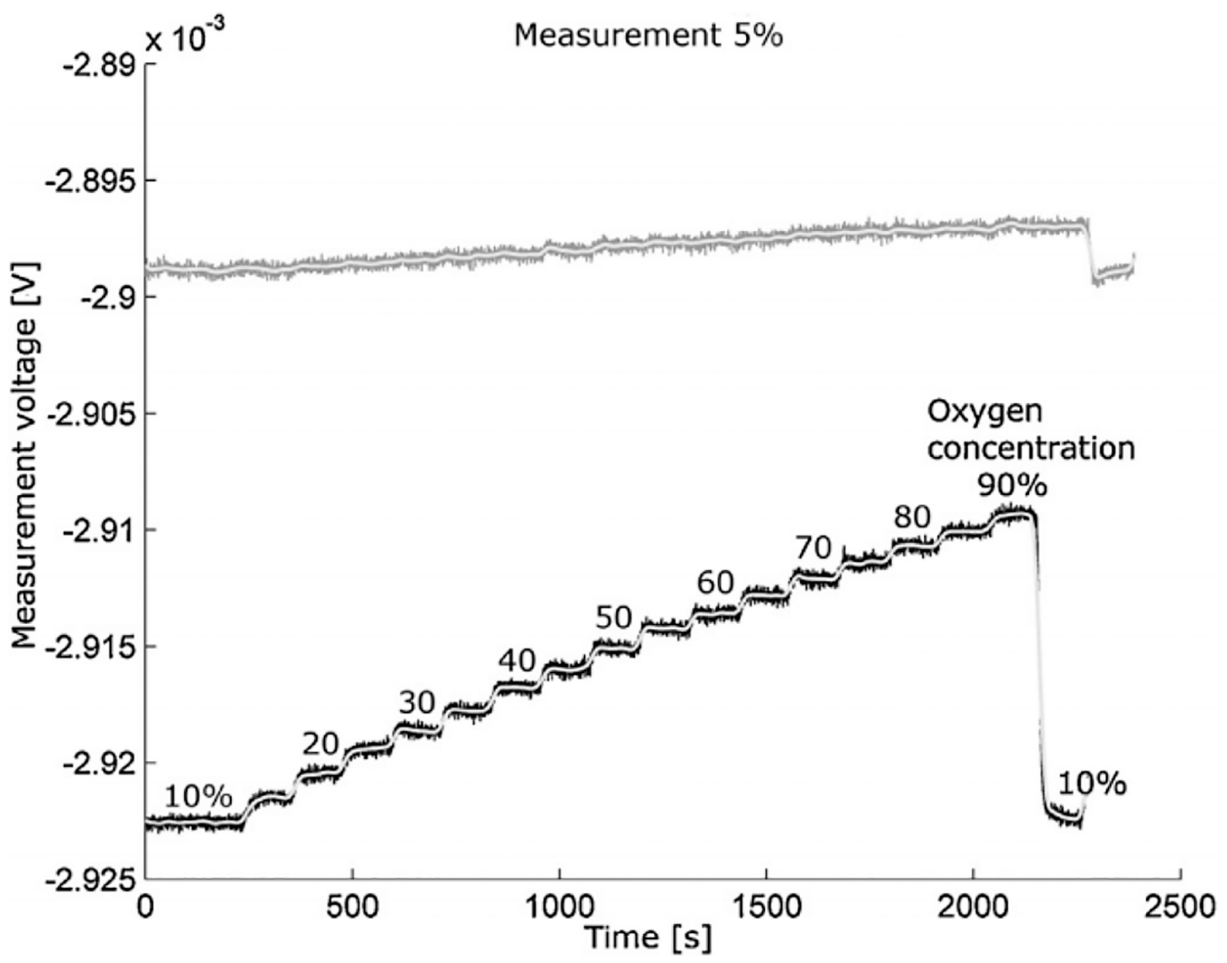
Sensor response as a function of the oxygen concentration in nitrogen. The gray line shows the reference measurement without the magnetic field. Reprinted with permission from ref (4). Copyright 2013 Elsevier.

Figure 15 shows the sensor response as a function of the oxygen concentration in nitrogen.

In Figure 15, the sensor response noise of 1% O_2_ and the signal drift due to changes in gas flow rate are observed. Because thermoanemometers are used to measure the gas flow velocity, the response depends on the thermal conductivity and heat capacity of the gas mixture. However, after appropriate signal processing, the sensor may be sufficient in applications that do not require high precision of measurement.

### Oxygen Sensors with Magnetic Field Strength Measurements

Paramagnetic substances increase the magnetic field induction, and, although the change is small, this phenomenon can be used to determine oxygen concentration in the presence of diamagnetic gases. The greatest difficulty with such measurements is the determination of a very small change in Δ**B** against the large constant component **B**. To estimate Δ**B**, let us assume that the sensor has a strong magnetic field with an induction of *B* = 1 T, which can be produced by large magnets made of rare earth oxides. If we introduce pure oxygen into the field area under a pressure of 1 atm at 298 K, then, according to eq 2, the change in induction with respect to nitrogen will be Δ*B* ≈ χ_O2_*H* = 1.8 μT.

Many types of sensors can be used to measure the strength of the magnetic field, the operation of which may be based on, among others, the Hall effect, giant magneto-resistance (GMR), anisotropic magneto-resistance (AMR), tunneling magneto-resistance (TMR), and giant magneto-impedance (GMI). A good review of new materials and possible mechanisms of giant magneto-resistance is described in ref (52). Moreover, interesting two-dimensional magnetic materials were recently developed. They have unique functions as the electric field control of a magnetic phase and the anomalous spin Hall effect.^53^ All of them are based on the change in the electrical properties of a material when an external magnetic field is applied. According to other work,^54^ the lower measurement limit of the GMI, TMR, and AMR sensors is below 1 nT, while for the GMR and Hall sensors it is about 1 μT or more. Hall sensors are preferably used at higher magnetic field values because they show no saturation effects in contrast to magnetoresistors (MRs). However, the detection of changes in the magnetic field induction by oxygen is at the limit of the measurement capabilities of Hall sensors. For this reason, oxygen sensors based on the absolute measurement of the magnetic field by means of Hall sensors have not been put into practice, although there are patents describing their operation.^55−57^

The relative orientation of the measured magnetic field vector with respect to the Hall sensor chip is perpendicular, and for MR sensors it is parallel. If the MR sensor is positioned perpendicularly to the magnetic field force lines, its indications will be zero. This fact can be used to measure the slight transverse fluctuations in field strength caused by the interaction of paramagnetic oxygen molecules with an external magnetic field. One of the ways of implementing this idea in practice is presented in another paper.^58^

**Figure 16:**
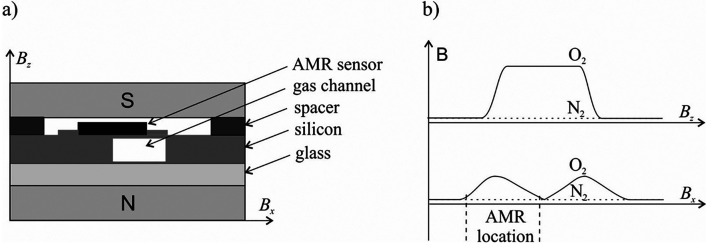
(a) Cross section of the sensor with AMR device. (b) Value of the magnetic field perpendicular to the channel.^58^

The principle of operation of the micro paramagnetic oxygen sensor described in the paper cited above is based on the deflection of a magnetic field in the vicinity of a gas channel. The sensor (Figure 16) consists of a silicon body placed on a glass substrate. The gas channel is etched into the silicon, and an AMR sensor is placed on one of its side walls. The whole body is located between the poles of a permanent magnet producing a flat magnetic field in the *z* direction. If diamagnetic gas (e.g., nitrogen) is present in the channel, the *B*_*z*_ component of the magnetic field is constant and the *B*_*x*_ component is zero. The orientation-sensitive AMR device is set up to measure only the *B*_*x*_ component of the magnetic field, so the signal is also zero. In the presence of oxygen, there is an increase in the *B*_*z*_ value around the channel and the *B*_*x*_ component appears, which is the gradient of *B*_*z*_ in the *x* direction (*B*_*x*_ = d*B*_*z*_/d*x*). The value of this component, which depends on the oxygen concentration in the gas channel, is measured by the AMR sensor.

The two-dimensional simulation of sensor operation was conducted with FEMM (Finite Element Method Magnetics) software.^58^ A constant magnetic field of 20 kA/m was applied to the area including the measurement channel with a geometry of 200 × 400 μm, and the perpendicular magnetic field at a distance of 100 μm outside the measurement channel was evaluated. The results of the simulation showed that the expected signal was approximately 2 nT.

The oxygen sensor was tested with oxygen/nitrogen mixtures. With a magnetic field (0.6 T) applied, 20% concentration steps from 0% to 100% of oxygen/nitrogen were observed with a change in the output signal of about 150 nV.

**Figure 17:**
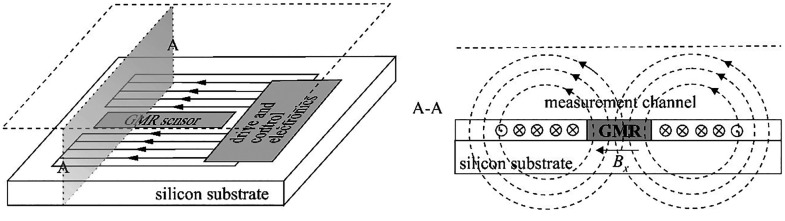
Scheme of the construction and principle of operation of an oxygen sensor with the giant magneto-resistance (GMR) device.^59^

A similar concept of measurement can be found in a patent,^59^ but the design solution itself is different (Figure 17).

In this case, the oxygen sensor comprises a GMR device, a magnetic field generator arranged to generate a magnetic field overlapping the GMR device, and an examination region. A component, *B*_*x*_, of the magnetic field, dependent on the oxygen concentration in the examination region, is detected by the GMR.

In the absence of oxygen in the examination region, the symmetry of the system causes the magnetic field to be oriented transversely to the planar magnetic field sensor. The spin-valve type of GMR device is sensitive to the magnetic field component *B*_*x*_ and is insensitive to other magnetic field components. As the oxygen concentration in the examination region increases, oxygen molecules align (in a statistical sense) with the magnetic field and strengthen it. This perturbation of the magnetic field introduces an asymmetry in the magnetic field that includes a perturbation magnetic field component, *B*, oriented along the *x*-direction, as shown in Figure 17. The GMR device detects and measures the perturbation magnetic field component *B*_*x*_. The measured in-plane component *B* is proportional to, or at least monotonically increasing with, the oxygen concentration in the examination region.

Another physical mechanism that makes it possible to measure changes in *B*, with the required accuracy, is laser interferometry. However, this method is difficult to apply due to the enormous sensitivity of the system to mechanical disturbances.

**Figure 18:**
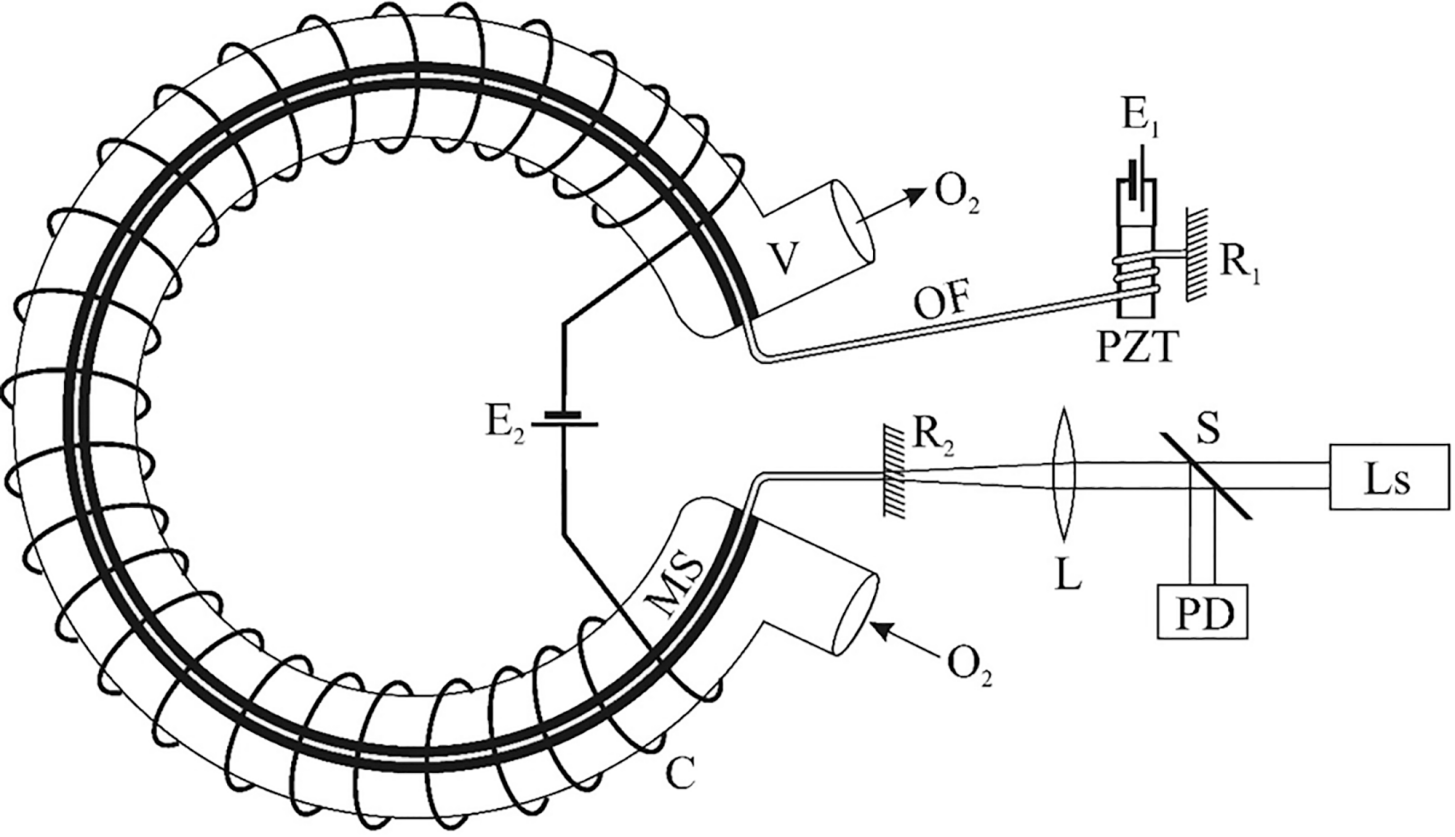
Fiber-optic magnetostriction sensor design.^60^

In another study,^60^ the authors presented the conceptual oxygen analyzer based on a phase sensor modulator detecting the change in the optical path length of a light flux in the signal arm of a fiber-optic interferometer (FOI). The change in the optical path is due to a distortion of the magnetostrictive material that is attached to the FOI signal fiber. An example of such a sensor design is shown in Figure 18.

The sensor consists of a toroidal measuring vessel (V), to which a strip of magnetostriction material (MS) is attached with a closely adjacent optical fiber (OF) loop. The OF loop together with the mirrors, *R*_1_ and *R*_2_, at their ends form an FOI. Dielectric coatings are used on the ends of the optical fiber to increase the reflectance (*R*) and thus ensure the required FOI quality factor. An electric coil, powered by a voltage source E_2_, is wound on the outer surface of the measuring chamber to form a constant magnetic field, *H*_0_, inside the chamber. The piezo-corrector is used to adjust the FOI to the operating point (a phase φ_0_), which corresponds to the maximum value of d*R*/dφ. The other structural elements marked in Figure 18, such as the lens (L), beam splitter (S), photodetector (PD), and laser (Ls), are standard for fiber interferometry in sensing applications.

The authors carried out numerical evaluation of the sensor’s operation and found that if the upper boundary of the measured concentration is mainly determined by the mechanical stability of the sensor construction, then its lower boundary will depend to a considerable extent on the choice of the method for measuring small currents of a photodetector. They suggest that a bridge measurement method provides comparatively simple measurement of O_2_ concentration at a level of ≤370 ppm, which corresponds to the relative values of O_2_ in gas mixtures at the level of fractions of a percent.

### Sensors Measuring the Change in the Physical Properties of a Gas

The drawing of oxygen molecules into the magnetic field causes not only an increase in pressure but also a local change in many other physical properties of the gas, such as density and thermal conductivity, as well as changes in the speed of sound or the refractive index. There have also been attempts to use these effects to measure oxygen concentration.

**Figure 19:**
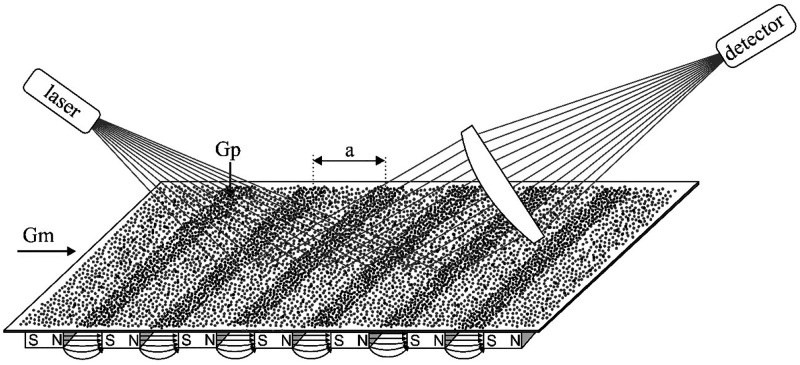
Principle of operation of a reflective gas diffraction grating.^61^

In one patent application,^61^ a magneto-optical measurement method was described. The idea is to group the oxygen molecules present in the gas mixture in the immediate vicinity of the sensor surface by means of a periodic magnetic field so that a diffraction grating is formed in the gas layer at the sensor surface. When the gas diffraction grating is illuminated, diffraction occurs, and by placing the light detector at a location corresponding to the diffraction angle, the intensity of the incident light can be measured. Intensity is a function of the refractive index change, which depends on the local magnetic field strength and the partial pressure of the paramagnetic gas. The advantage of this kind of sensor is the short response time and the long sensor service life. In addition, this sensor works without a reference gas and does not require a pump. The sensor has a small size and simple design and is not very sensitive to environmental disturbances. The principle of operation of such a sensor is shown in Figure 19.

The device (Figure 19) is made of a matrix of elongated magnetic elements placed periodically under the outer surface of the sensor. The magnetic elements are magnetized in such a way that their magnetic poles are located on the longer edges of the elements. The elements are arranged with defined gaps, and the neighboring magnets face each other with opposite poles. In each gap between the magnets, a magnetic field is created, which extends over the outer surface of the sensor, affecting the gas mixture there.

Paramagnetic gas molecules present in the gas mixture above the sensor surface move toward the longitudinal regions of the magnetic field from the magnetic elements below the surface. As a result, long, narrow, and shallow areas with a high concentration of paramagnetic gas are formed on the outer surface of the sensor. As the gas density in this regions increases, the refractive index also increases, and a phase difference of monochromatic light reflected from this regions is created, causing diffraction.

One patent application^61^ describes the many configurations of this apparatus, including a reflection and transmission diffraction grating composed of magnetic elements, various grating patterns, and methods of generating a magnetic field. Again, however, there are no experimental results confirming the practical usefulness of this method.

Patent applications^62,63^ describe oxygen sensors based on measuring changes in speed of sound and thermal conductivity. However, both of these methods are, by definition, nonselective because both the thermal conductivity and the speed of sound strongly depend on the gas composition. As a result, the effects arising from changes in the concentration of components accompanying oxygen may be many times greater than those resulting from oxygen paramagnetism. Moreover, they also have the problem of measuring very small changes in a physical quantity in the presence of a large constant component.

## Summary

Magnetic sensors are an important group of oxygen sensors, characterized by high measurement accuracy and durability. They are used mainly in areas where the credibility and reliability of the measurement are the most important, such as industrial process control or medicine.

There are many types of sensors that use the paramagnetic properties of oxygen, but the most common are “dumbbell” type, magnetoacoustic and magnetopneumatic sensors, or sensors using the principle of thermo magnetic wind. In industrial and laboratory research, dumbbells and magnetic wind sensors are mainly used. However, where a very small time constant of the device and differential measurement against the reference gas are required, for example, in medicine, magnetoacoustic and magnetopneumatic analyzers dominate.

With all paramagnetic sensors, the number of oxygen molecules per unit volume is measured. Therefore, when calculating the oxygen volumetric concentration according to the gas state equation, we must take into account the influence of temperature and pressure. In addition, the change in magnetic susceptibility with temperature must also be considered. For this reason, the measurement error of these instruments largely depends on ensuring stable measurement conditions, that is, temperature and flow control, as well as isolation of the measuring chamber from mechanical disturbances and is usually below 1% of the measurement range. The detection limit of paramagnetic analyzers is at the level of single ppm of O_2_. Table 2 lists the most important features of each type of oxygen analyzer.

**Table 2:** Comparison of Different Types of Paramagnetic Oxygen Analyzers

type of analyzer	magnetic field	measured physical effect	transducer	time constant^a^	detection limit^b^	comments	refs
dumbbell	constant	buoyancy force	optical, measuring the twist of the dumbbells	5–10 s at 100 mL/min	100 ppm	sensitive to vibrations, limited to stationary applications, many commercial devices	(9−11, 13−27)
dumbbell MEMS	constant	buoyancy force	optical, measuring the twist of the dumbbells	1.3 s	50 ppm	sensitive to vibrations, experimental	(12)
resonator	constant	pressure vibration	bimorphic resonator	0.1 s	n/a	experimental, no commercial devices, measurement range: 10–90% O_2_	(28)
magnetoacoustic with reference gas	alternating (100–200 Hz)	pressure variation	differential microphone	0.15 s	300 ppm	differential (reference gas required), sensitive to vibrations, applied in anesthesiology, many commercial devices	(29−37)
magnetoacoustic without reference gas	alternating (100–200 Hz)	pressure variation	microphone	<1 s	n/a	sensitive to vibrations, mainly patent literature	(38−40)
magnetopneumatic	alternating (several Hz)	pressure variation	thermoanemometric flowmeters in the pneumatic bridge	1.5–3 s	n/a	resistant to vibration, very good compensation of temperature and power fluctuations, many commercial devices	(41−43)
with magnetic wind	constant	magnetic convection of gas	thermoanemometric	5 s	70 ppm	sensitive to temperature and pressure changes, commercial devices available	(44−50)
with deflection of the gas stream	constant	deflection of the gas stream at magnetic field	thermoanemometric	<1 s	1%	MEMS, experimental, no commercial devices	(4, 51)
with Hall sensor	constant	increasing the magnetic field	Hall sensor	<1 s	n/a	MEMS, only patents, there are neither experimental nor commercial devices	(55−57)
with AMR sensor	constant	increasing the magnetic field	anisotropic magneto-resistance sensor	<1 s	>1%	MEMS, experimental, no commercial devices	(58)
with GMR sensor	constant	increasing the magnetic field	giant magneto-resistance sensor	<1 s	n/a	patent, there are neither experimental nor commercial devices	(59)
interferometric	constant	distortion of the magnetostrictive material	fiber-optic interferometer	n/a	370 ppm	enormous sensitivity to mechanical disturbances, experimental, no commercial devices	(60)
refractometric	constant	changes in refractive index	light detector measuring the diffraction angle on diffraction grating	<1 s	n/a	patent, there are neither experimental nor commercial devices	(61)
ultrasonic	alternating (low frequency)	changes in speed of sound	ultrasonic resonator	n/a	n/a	patent, there are neither experimental nor commercial devices	(62)
thermal-conductive	alternating	changes in thermal conductivity	thermopile or other temperature sensor	n/a	n/a	patent, there are neither experimental nor commercial devices	(63)

a The time constant of the analyzers depends on the measuring cell volume and the gas flow rate. In many types of sensors, the measurement of the physical effect itself is instantaneous, and the time constant results solely from the gas exchange rate in the measuring cell.

b Detection limit applies to experimental or commercial instruments and not to the physical effect itself.

By observing the reports presented in the scientific literature and the latest patent applications, two major trends in the development of paramagnetic oxygen sensors can be distinguished. The first is the miniaturization of known types of sensor designs. Examples include microphone sensors and inert gas displacement sensors manufactured using MEMS technology. The miniature analyzers produced so far have slightly worse parameters than their classic counterparts, but the very rapid development of MEMS technology allows for continuous improvement of their design. In the future, miniature, cheap, and commercially manufactured oxygen sensors will probably find applications in areas where they are currently used sporadically, for example, in household ventilation systems. In addition, sensors produced in the MEMS technology, due to their miniature size and low energy consumption, will be able to be mounted in everyday objects such as, for example, mobile phones or watches.

The second visible direction of development is the construction of sensors with a different principle of operation than commonly used devices. Novel ideas concerning the measurement of oxygen concentration by means of various physical effects resulting from its paramagnetic properties are constantly appearing in the literature. These include sensors that use gas flux deflection and changes in magnetic field strength, refractive index, thermal conductivity, and speed of sound. The greatest advantages of this type of solutions may be the simplicity of construction, reliability, and insensitivity to shocks resulting from the lack of moving mechanical parts. These sensors are currently at the stage of laboratory research, and their continuous development and systematic improvement of metrological parameters should be expected. In the future, these devices will probably find application in measurements carried out in harsh environments, exposure to vibrations, shocks, and noise, where the use of traditional designs is problematic.

## Vocabulary

magnetic buoyancy force: buoyancy force acting on the nonparamagnetic body (gas molecules) as a result of the local increase in the partial pressure of a paramagnetic gas caused by an external magnetic field
Pauling cell oxygen sensor: a classic design of a paramagnetic oxygen sensor in which two nonparamagnetic gas-filled glass spheres are connected to each other like dumbbells and mounted on a rotating suspension between poles of strong permanent magnet; the oxygen contained in the analyzed gas accumulates between the poles of the magnet and, pushing out the nonparamagnetic spheres, causes the dumbbells to rotate in proportion to the oxygen concentration
magnetoacoustic sensor: a sensor in which the paramagnetic gas concentration is measured by measuring the amplitude of gas pressure vibrations in an external, alternating magnetic field; the frequency of the magnetic field corresponds to the acoustic band, and oscillations of the pressure are measured with a microphone
magnetopneumatic sensor: a sensor in which the paramagnetic gas concentration is measured by measuring the gas flow rate in the pneumatic bridge; the magnetic field locally increases the pressure of the flowing gas in one of its arms, causing the bridge to be unbalanced and the flow to appear on its diagonal
magnetostatic sensor: a sensor that uses a constant magnetic field
magnetodynamic sensor: a sensor that uses an alternating magnetic field
